# Electrochemical impedance spectroscopy applied to microbial fuel cells: A review

**DOI:** 10.3389/fmicb.2022.973501

**Published:** 2022-07-22

**Authors:** Hui Wang, Xizi Long, Yingying Sun, Dongqi Wang, Zhe Wang, Haiyu Meng, Chunbo Jiang, Wen Dong, Nan Lu

**Affiliations:** ^1^Department of Municipal and Environmental Engineering, Faculty of Water Resources and Hydroelectric Engineering, Xi’an University of Technology, Xi’an, China; ^2^International Center for Materials Nanoarchitectonics (WPI-MANA), National Institute for Materials Science, Tsukuba, Japan; ^3^Technology Innovation Center for Land Engineering and Human Settlements, Shaanxi Land Engineering Construction Group Co., Ltd., and Xi’an Jiaotong University, Xi'an, China

**Keywords:** electrochemical impedance spectroscopy, biofilm capacitor, microbial fuel cell, electroactive bacteria, electron transfer, distribution of relaxation time

## Abstract

Electrochemical impedance spectroscopy (EIS) is an efficient and non-destructive test for analyzing the bioelectrochemical processes of microbial fuel cells (MFCs). The key factors limiting the output performance of an MFC can be identified by quantifying the contribution of its various internal parts to the total impedance. However, little attention has been paid to the measurement conditions and diagrammatic processes of the EIS for MFC. This review, starting with the analysis of admittance of bioelectrode, introduces conditions for the EIS measurement and summarizes the representative equivalent circuit plots for MFC. Despite the impedance from electron transfer and diffusion process, the effect of unnoticeable capacitance obtained from the Nyquist plot on MFCs performance is evaluated. Furthermore, given that distribution of relaxation times (DRT) is an emerging method for deconvoluting EIS data in the field of fuel cell, the application of DRT-analysis to MFC is reviewed here to get insight into bioelectrode reactions and monitor the biofilm formation. Generally, EIS measurement is expected to optimize the construction and compositions of MFCs to overcome the low power generation.

## Introduction

Traditional wastewater treatment technologies require a large amount of energy to remove the chemical oxygen demand, nitrogen, and phosphorus (Smith and Liu, 2017; Awad et al., 2019). This energy consumption results in significant carbon emissions. The microbial fuel cell (MFC) technology, benefitting from treating wastewater and recovering energy simultaneously, has developed their basic construction rapidly over the past 20 years. A biofilm attached to an anode transfers electrons through transmembrane heme proteins to the anode electrode, and the final electron acceptors, such as oxygen and nitrate at the cathode, react with these electrons to form a closed circuit (Logan et al., 2006). However, the inherit large resistance and their distinct characteristics of MFC with bioelectrode necessitate an evaluation and diagnosis of their performance and operating status (Rabaey et al., 2010; Mashkour and Rahimnejad, 2015). To date, the high internal resistance of an MFC limits its power output (Rabaey and Verstraete, 2005), which is from the extracellular electron transfer of the electroactive bacteria (EAB; Zheng et al., 2020). Since the EAB thrives under mild environmental conditions with a stable temperature, neutral pH, and sufficient nutrients, the electrolytes in MFCs must be supplemented with buffers and trace elements to maintain the metabolism of EAB (Li et al., 2013; Lee et al., 2016; Pandey et al., 2016), inferring that MFCs cannot use high-concentration, high-conductivity, or toxic solutions, and leading the lower performance of MFCs compared with chemical fuel cells. Moreover, in order to maintain a stable high current output, EAB cells are stacked into biofilms with thicknesses of more than 10 μm at the anode. However, such a thick biofilm could hinder electron transfer to the electrode interface, organic carbon diffusion to the EAB, and result in the accumulation of protons (Artyushkova et al., 2015). Accordingly, the performance of bioelectrochemical systems, such as MFCs, microbial electrolytic cells, and microbial desalination cells, is limited. Although research has been conducted on the optimization of structural configurations and modification of MFC materials (Zhang et al., 2014; Hindatu et al., 2017; Peiravi et al., 2017), their performance is far from commercial scaling up (Santoro et al., 2017). Consequently, it is vital to quantify key factors limiting the performance of MFCs (Rismani-Yazdi et al., 2008).

**Figure 1:**
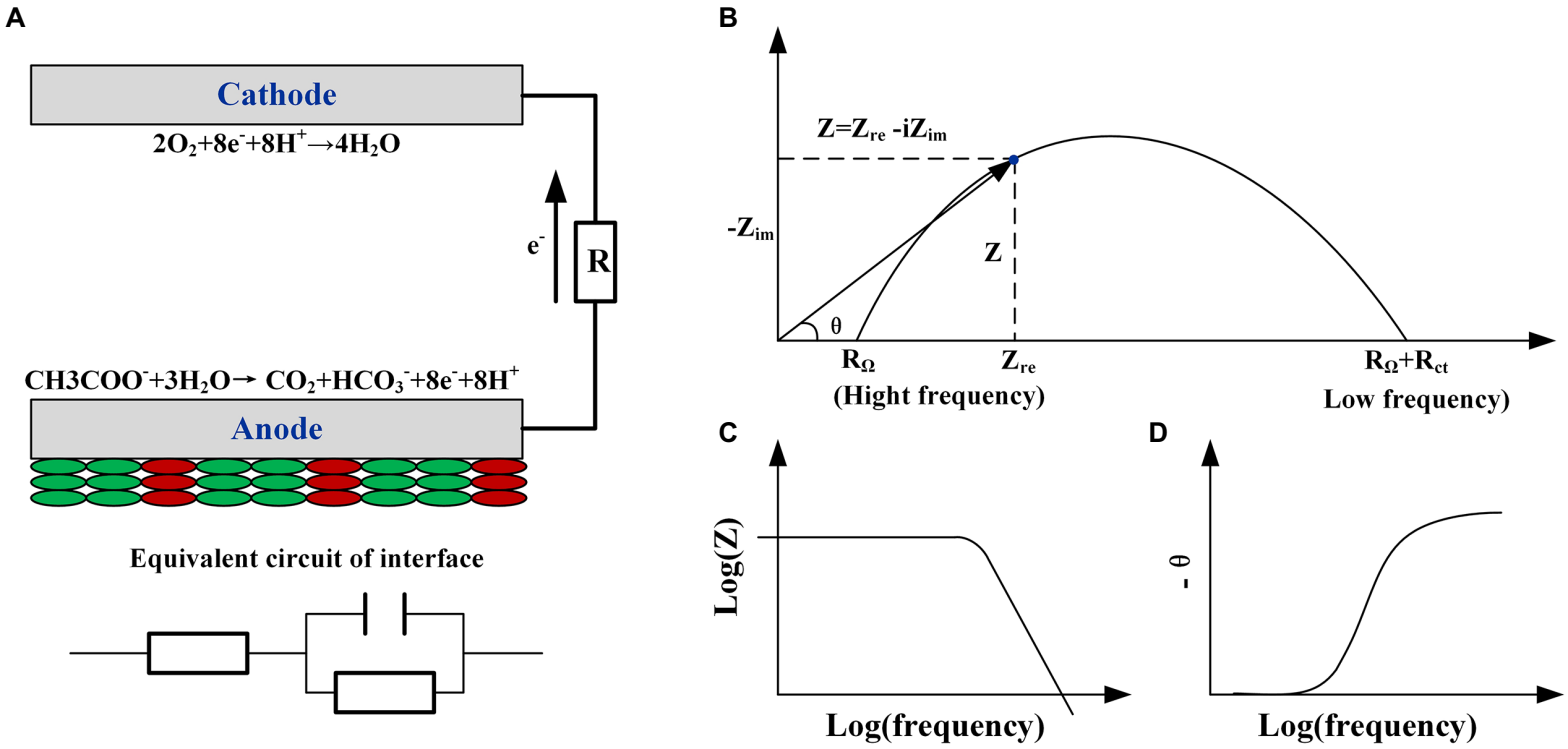
**(A)** The schematic of microbial fuel cell’s reaction and the equivalent circuit of the anode interface R(RC). **(B)** Nyquist, **(C)** BODE, and **(D)** phase angle plots of the circuit.

Electrochemical impedance spectroscopy (EIS) is the most suitable technique for identifying the limitations in various parts of MFC in terms of impedance (Figure 1A; Manohar et al., 2008b). Researchers have analyzed electron transfer processes under different conditions including the MFC configuration, cathode and anode biofilm, and electrode materials, according to the BODE and Nyquist diagrams (Figures 1B,C; Sekar and Ramasamy, 2013; Kashyap et al., 2014). Then, different BODE and Nyquist diagrams, obtained from EIS used in various MFC studies with different configurations and application ranges, are classified. Meanwhile, the origin and meaning of various figures in the impedance spectrum are explained. Despite the impedance part, the imaginary part in the EIS, representing the capacitance, is reviewed. Finally, a new analysis tool for the EIS-distribution of relaxation times (DRT) is introduced to simplify the EIS analysis process, improve the analysis, and allow EIS to play a better role in the analysis of MFCs.

## Application principle of EIS in bioelectrodes

Measurement methods such as alternating current (AC) EIS, cyclic voltammetry (CV), linear sweep voltammetry, and Tafel plots are widely used in MFC research (Zhao et al., 2009). In contrast to techniques employing a wide range of control potentials to obtain a current, EIS limits the amplitude of the perturbation voltage to less than 10 mV and uses a feedback linear signal to measure the frequency domain over a wide range (Ciucci, 2019). Electrode processes that satisfy the causality conditions and remain relatively stable during disturbances can be investigated using EIS. The electrochemical impedance spectrum can be fitted and analyzed using the equivalent element method. However, the impedance model is a typical black box, and the equivalent circuit formed by arranging different electrical components can fit the same set of impedance data. Therefore, a specific and practical equivalent circuit can be obtained by investigating an MFC in combination with the reaction kinetics of MFC electrodes (Chang and Park, 2010).

Since the exchange current density is small, MFCs are usually regarded as quasi- and ir-reversible reactions, which indicates a Nernst relationship cannot be established between the electrode potential and activity of the surface reactive species (regarded as a protein with redox activity). The current is related to the electric potential, surface state variables, and reactive species as follows (Bard et al., 2022):


(1) $$I_{f}=f\left(E,X_{i},C_{j}\right)\;\;i=1,2,n\;\;j=1,2,m$$

Using the Taylor expansion from the linear conditions, we can obtain the following:


(2) $$\Delta I_{f}={\left(\frac{\partial I_{f}}{\partial E}\right)}_{ss}\Delta E+\overset{n}{\underset{i=1}{\sum }}{\left(\frac{\partial I_{f}}{\partial X_{i}}\right)}_{ss}\Delta X_{i}+\overset{m}{\underset{j=1}{\sum }}{\left(\frac{\partial I_{f}}{\partial C_{j}}\right)}_{ss}\Delta C_{j}$$

where *I_f_* is the current, *ss* indicates that the electrode reaction is in a steady state, E denotes the potential disturbance, $X_{i}$ indicates the electrode surface state variable that can influence the reaction rate of the electrode surface (such as the biofilm thickness and coverage area), and $C_{j}$ represents the surface activity of reactant j (correlated to the surface concentration of cytochrome *c*), and Δ represents the corresponding disturbance; moreover,


(3) $$\Delta E=E-{\left(E\right)}_{ss}$$

(4) $$\Delta X_{i}=X_{i}-{\left(X_{i}\right)}_{ss}\;\;i=1,2,\cdots ,n$$

(5) $$\Delta C_{j}=C_{j}-{\left(C_{j}\right)}_{ss}\;\;j=1,2,\cdots ,m$$

Further derivation produces the resistance condition:


(6) $$Y_{f}=\frac{1}{R_{t}}+\sum m_{i}\frac{\Delta X_{i}}{\Delta E}+\sum p_{j}\frac{\Delta C_{j}}{\Delta E}$$

where $Y_{f}$ is admittance $1/R_{t}={\left(\partial I_{f}/\partial E\right)}_{ss}$ , $R_{t}$ denotes the electron transfer internal resistance, and $m_{i}={\left(\partial I_{f}/\partial X_{i}\right)}_{ss}$ , $p_{j}={\left(\partial I_{f}/\partial C_{j}\right)}_{ss}$ .

These equations (1–6) serve as the basis for the actual equivalent circuit of the Faraday process for complex bioelectrode reactions. The impedance distribution under different conditions can be obtained by simplifying the real conditions, which can be used to explain the actual circuit process. Notably, these formulas clearly demonstrate that the internal resistance of electron transfer is not equal to that of polarization resistance. In fact, most electrode reactions in MFCs are slow, and the internal resistance of polarization is greater than that of electron transfer. The slope of the polarization curve of an MFC reflects the relationship between the output voltage and current under steady-state conditions; which only represents the polarization internal resistance. The EIS can classify the polarization internal resistance as electron transfer, ohmic, or diffusion internal resistances, and these are beneficial for optimizing interface impedance and improving the electron transfer rate.

## Polarization conditions for EIS testing

EIS measurements are typically performed under stable open-circuit conditions (Manohar et al., 2008a; Kim et al., 2009; Wang et al., 2009). However, experiments have revealed that impedance spectra significantly change at different potentials (Ramasamy et al., 2008; Zhu et al., 2013; Rodenas Motos et al., 2015). Researchers have performed EIS measurements during the potentiostatic cultivation of anode biofilms at different polarization potentials. The results indicated that the resistance decreased to a minimum as the potential approached the midpoint potential of the catalytic wave obtained using CV measurements from a negative potential (Marsili et al., 2008); the resistance then increased with the increase in potential (Jung et al., 2011; Rossi et al., 2020). Although electrical potential stimuli may influence the gene expression of EAB, changes in the electrical potential within a short time are inadequate for the production of obvious effects on microorganisms (Zhu et al., 2014). Hence, this temporary potential change may not influence the microbial community structure (Kim et al., 2017). Li et al. believed that this change may be related to the cytochrome c protein involved in electron transfer (Li et al., 2014). The electron “jump” during the redox process between the active centers of the porphyrin structure in the cytochrome c protein is considered the only method for extracellular electron transfer. The oxidation state/reduction state ratio of cytochrome *c* varies with corresponding changes in the electrode potential, which affects EIS measurements (Li, 2014). In their study on the self-recovery of stacked MFCs after voltage inversion by EIS, Kim et al. discovered that temporary voltage inversion was caused by degrading reaction kinetics at the anode relative to the cathode. The anodic voltage reversal may be due to a temporary decrease in the anodic electrode performance, and this change may be related to a change in the biofilm capacitance (Kim et al., 2017).

However, this explanation is not applicable to EIS measurements of MFCs in the open-circuit state because the anode and cathode, with overpotential under closed-circuit conditions, are affected by the changes in external resistance and key elements affecting the impedance. Therefore, we believe that the open circuit is not the optimal measurement condition. Qiu et al. (2015) investigated the EIS of abiotic cathode MFCs under different conditions and discovered that certain polarization conditions demonstrated a greater impact on the cathode internal resistance, particularly the low-frequency diffusion part. The study revealed that an MFC cathode uses stainless steel as the diffusion backing and adds 1.56 mg/cm^2^ of carbon black; the diffusion internal resistances obtained using EIS differed significantly from each other: 72.28, 37.67, and 12.41 Ω for polarization conditions of 0.1, 0, and − 0.1 V, respectively. That is, higher the degree of polarization adopted by the EIS, lower the internal resistance (Qiu et al., 2015). The closed-circuit voltage is the operating voltage at which the MFC produces current. Rodenas Motos et al. (2015) raised doubts concerning the suitability of the internal resistance measured under open-circuit conditions for the actual internal resistance during operations (Rodenas Motos et al., 2015). Jung et al. (2011) compared EIS test results of a hydrogen fuel cell, consequently concluding that the polarization state under the working state was superior (Freguia et al., 2007; Jung et al., 2011). Therefore, a selection of polarization conditions under the working state is more representative because the impedance obtained using EIS of MFCs demonstrates evident differences under different polarization conditions.

## Analysis of EIS spectra

Typically, EIS is performed under an AC voltage of 5 or 10 mV over a frequency range of 100 kHz to 1 MHz (or lower). Information regarding the real part (resistance) and imaginary part (capacitance) corresponding to the frequency can be obtained, and thereafter, a Nyquist complex plane diagram containing the real and imaginary parts, Bode diagram describing the change in total impedance with frequency, and phase angle with frequency change can be created (Figures 1B–D). The angle diagram (Figure 1D) contains complex information such as the number of state variables of the reaction and time constants. The number of state variables corresponds to the number of potentials, material activity, and biofilm surface coverage (Equation 6). The distribution and number of time constants (product of the capacitance and resistance) directly reflect the number of state variables. Two methods are typically used when EIS is applied to MFCs: the whole-cell test or electrode test. The working electrode is typically the anode in whole-cell testing, and the counter and reference electrodes are wired to the cathode. However, owing to the similarity between the time constants of the cathode and anode reactions, the impedance spectrum of the whole-cell cannot clearly distinguish different responses of the cathode and anode. For the EIS test of a single electrode, the ohmic, electron transfer, and diffusion internal resistances represent the primary components of the internal resistance of the MFC under working conditions (polarization). However, researchers may find it easy to mechanically match equivalent circuit elements to the internal resistances when fitting these three internal resistances without considering the influence of actual electrode and biofilm compositions on the EIS response. Therefore, the impedance spectrum should be classified (including the arcs formed by different frequency points in the Nyquist diagram, number of peaks in the BODE diagram, and relationship between their positions and frequencies) and combined with the actual MFC electrode process to obtain a result that is consistent with the actual situation.

### Single capacitive reactance arc formed by different frequency points on the Nyquist diagram

Electrodes are usually simplified and are equivalent to Randles circuits in EIS studies (Harrington and van den Driessche, 2011). Randles circuits contain a non-conductive interfacial capacitance that functions as an electric double layer and electron transfer pathway. Such a circuit represents the most simplified electrode equivalent circuit, which corresponds to a standard semi-circular arc composed of different frequency points on a relatively simple single-arc Nyquist diagram. This implies that a single reaction process may occur in the MFC. Therefore, the Randles equivalent circuit R_e_(R_t_C_dl_) can be used for simulation, where R_e_ and R_t_ represent the ohmic and electron transfer internal resistances, respectively, and C_dl_ denotes the electric double-layer capacitance. Because the electrodes used in MFC research are essentially unpolished, they possess a rough surface and an uneven three-dimensional direction, resulting in significant differences between the imaginary impedance of most patterns and capacitor circuit characteristics (Orazem and Tribollet, 2008; Sindhuja et al., 2019). Randles circuits are rarely employed in complex MFC systems involving mass transfer, biochemical reactions, protein electron transport, and reduction reactions (usually oxygen reduction). Therefore, a constant phase element (CPE) is often used to meet the fitting requirements (Wen et al., 2010; Xu et al., 2010; Du et al., 2014; Hidalgo et al., 2015; Hou et al., 2015).

A typical MFC consists of an anode, which is usually a carbon electrode with improved biocompatibility, and a cathode, which is usually an air-diffusion cathode with a platinum catalyst. Hutchinson et al. (2011) conducted EIS tests on a classic single-chamber air cathode MFC, where the anode Nyquist diagram was a single arc, and the electron transfer resistance was only 18 Ω after fitting the equivalent circuit. The MFC cathode is routinely considered the primary factor limiting the power generation performance of MFCs. Potassium ferricyanide is determined to be a satisfactory electron acceptor that can significantly improve the electricity production performance of two-chamber MFCs. Accordingly, when potassium ferricyanide serves as a cathode electron acceptor, the different frequency points in the Nyquist diagram obtained from the cathode EIS test form a single half-arc. In addition, Gadhamshetty et al. (2013) tested the effects of three pretreatment methods on the impedance of different electron donors; consequently, the impedance was found to be a single half-arc when potassium ferricyanide was used as the electron acceptor (Lepage et al., 2012; Hou et al., 2015). Lepage et al. (2012) compared the MFC performance for carbon cloth and graphite-coated anodes using a cathode filled with 50 mM of potassium ferricyanide as the electron acceptor. The cathode Nyquist plot was a single arc, and the electron transfer internal resistance was 12.5 Ω. Fraiwan et al. (2014) discovered that the Nyquist plot of a cathode with a volume of 57 μl containing potassium ferricyanide and a buffer solution also exhibited a single arc. A single-arc Nyquist diagram was also obtained when 2000 mg/l of chloroauric acid was used as the electron acceptor for the EIS test of a whole-cell and single electrode (Choi and Hu, 2013). The change in arc shape at a certain frequency is often used to explain the change in electron transport under certain conditions. The internal resistance decreased significantly from 1 to 113 days, and the impedance plot in the low-frequency band, which initially indicated the diffusion limitation, gradually collapsed into a circular arc (Aaron et al., 2010). In conjunction with the first example, the graph may reflect a diffusion-controlled change in an electron transport-limited process. The experiment also discovered that an increase in the flow rate and ionic strength caused a decrease in the MFC internal resistance and improved the power output within the test range (Aaron et al., 2010).

The aforementioned EIS tests were performed under different MFC configurations, electrode materials, operating conditions, and research purposes. The Nyquist plots of single arcs formed at different frequency points suggest that these MFC electrode processes may have similar properties. The results indicate that (1) single arcs mostly occur in single electrode tests rather than whole-cell EIS measurements, and single electrode tests reduce the electrode reaction with a different time constant; (2) when oxygen is used as the electron acceptor, the general air cathode reaction is slow even when aeration is used. When potassium ferricyanide is used as the electron acceptor, the reaction rate of the cathode electrode and MFC power are significantly improved. Air cathodes are prone to diffusion limitations, and high concentrations of potassium ferricyanide or chloroauric acid can thin the diffusion layer. A common feature of single arcs is that they all use high-performance cathode and anode MFCs, and their Nyquist patterns are observed to often correspond to a single arc. The overlapping of Nyquist plot impedance values within an arc is easily overlooked. Multiple experiments have demonstrated that the whole-cell has a single arc similar to that of the anode or cathode, which suggests that multiple reaction processes are not easily distinguishable owing to the close proximity of time constants. When the reaction time constants of the anode and cathode are similar, the arcs of the Nyquist diagram may overlap and still appear as a single arc with poor symmetry (Hou et al., 2015). This implies that the single arc response obtained by impedance measurements of a single electrode may be the result of superposition of multiple responses under special circumstances; therefore, the fitting results are sometimes disappointing (Lepage et al., 2014; Hidalgo et al., 2015).

**Figure 2:**
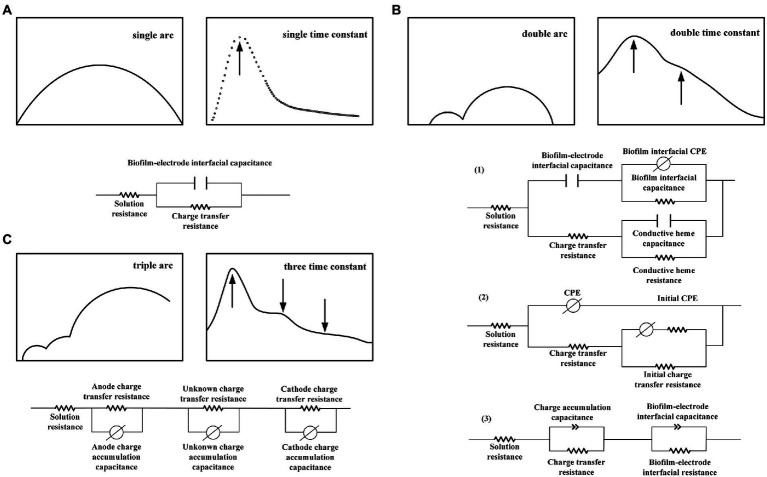
Representative impedance equivalent circuit with **(A)** single-time constant, **(B)** dual-time constant, and **(C)** three multiple-time constants.

However, a single arc does not imply that the electrode reaction is a single process. Carbon brushes, carbon cloths, and foamed carbons with large specific surface areas can exhibit the characteristics of a single-polarization internal resistance on an electrode under suitable conditions (high concentrations of nutrient solution and a good buffer). For a soil MFC, the mass and electron transfer processes are relatively slow, and the system impedance value is high. Although the Nyquist plot maintains a single arc shape, the data points are more cluttered under low-frequency conditions, suggesting that numerous reactions may occur within the soil MFC. Therefore, a summary of the EIS spectra of MFCs is necessary. Notably, the equivalent circuit of a single time constant does not consist of only a single reaction. However, the change in the MFC power output performance can be appropriately and convincingly characterized by a comparative study of the change in impedance under different parameter conditions. When the arc is complete and symmetrical, that is, when only a single time constant is exhibited, the internal resistance, which is in parallel with the capacitor in the Randles equivalent circuit, can be regarded as a general polarization internal resistance rather than an electron transfer internal resistance. In a poorly symmetrical impedance arc, BODE plot trends can be observed and compared to the time constants before performing an equivalent circuit simulation. Nonetheless, a consideration of the actual physical process is more meaningful when analyzing the whole-cell and performing circuit fitting based on actual electrode surface conditions. By establishing a physical model that corroborated the actual electrode process, Li et al. (2014) established an equivalent circuit to study the long-range electron transfer strategy of a *Geobacter sulfurreducens* PCA pure bacterial biofilm. They believed that three primary mechanisms of electron transfer were present in PCA biofilms: electron hopping between heme porphyrin rings (Kheme-heme), heme, and conductive nanowire conjugated groups (Kheme-pilus), and conjugated groups of conductive nanowires (Kpilus-pilus). Therefore, they proposed an equivalent circuit combining the aforementioned three processes (Figure 2). An electrode potential of 0.1 V (vs. SHE) produced a single arc Nyquist diagram for the PCA biofilm, an internal resistance for the interface electron transfer of 2.305 KΩ/cm^2^, and an internal conduction capacitance in the biofilm of 20.30 μF/cm^2^, which suitably matched the EIS test results. Therefore, by further observing the Nyquist and BODE diagrams according to actual physical processes, a more reasonable EIS equivalent circuit can be proposed to quantitatively analyze the impedance characteristics.

### Multiple capacitive reactance arcs composed of different frequency points in the Nyquist diagram

A graph containing multiple arcs or phase angle peaks is more complex than a single arc and conveys more information. The independent capacitive response of two electrodes is prone to exhibiting multiple arcs for whole-cell measurements. Studies on MFC electron transport and rate-limiting steps using impedance arcs at different locations with high, middle, and low frequencies have been widely reported (Ramasamy et al., 2009). For example, researchers have compared the MFC power generation effects using potato residue and glucose as carbon sources by evaluating the steps corresponding to the reactions through EIS; consequently, they speculated that the difference in arcs corresponding to low-frequency points in the Nyquist diagram may be owing to lower oxidation rates of the substrates. The intermediate-frequency arcs in the Nyquist diagram correspond to the electrochemical responses of the redox mediators released by substrate oxidation, whereas the high-frequency arcs are attributed to fast redox processes associated with metal salts. In addition, we conclude that the following three types of MFCs exhibit multi-arc Nyquist plots for EIS measurements:

1. Modified MFC electrodes. Researchers coated graphite onto stainless steel and carbon cloth to form an anode. The Nyquist diagram of the anode electrode changed from a single to double arc; the increased time constant corresponded to the electrochemical response of the coating. Other coatings such as graphene, carbon nanotubes, activated carbon, and composite electrodes may exhibit multiple arcs. The electrochemical reaction of the modified electrode may occur simultaneously at multiple reaction interfaces, such as electrode/electrolyte and new material/electrolyte, which will inevitably lead to an increase in the number of response arcs. This indicates that EIS is an important method for assessing the modification of MFC electrode materials.
2. A clear diffusion limitation exists in MFCs. In the Nyquist diagram of the MFC air cathode, arcs corresponding to electron transfer at high frequencies and diffuse arcs (or straight lines with a phase angle of 45 °) composed of frequency points with a constant phase angle are often observed, which is strongly related to the diffusion layer formed at the electrode interface (Wen et al., 2010; Sevda et al., 2013; Jadhav et al., 2014). Hidalgo et al. (2015) produced a whole-cell Nyquist plot with distinct diffusion features. After fitting the frequency point to the equivalent circuit, the impedance of the anode diffusion process corresponding to the low-frequency impedance was found to be three orders of magnitude higher than the impedance of the electron transfer process corresponding to the high frequency. This conclusion indicates that the anode diffusion process (corresponding to a low frequency) contributes to the majority of the MFC impedance, which is the rate-limiting step of the MFC.
3. MFC scale-up reactor. From an EIS perspective, MFCs with poor power generation and large MFCs often have reactant diffusion limitations (Jadhav et al., 2014; Flimban et al., 2019). While operating these devices, the non-electrochemically active bacteria in the biofilm rapidly proliferate, causing the biomass to increase of electricity production. Other colonies may also crowd the reaction electrode surface, and extracellular polymeric substances formed by these colonies may hinder the diffusion of protons to the electrode surface (Long et al., 2019). The electron donors required by microorganisms must diffuse before they can be used. A thick diffusion layer is formed when the diffusion is poor, and this limits the metabolism of microorganisms. In addition, studies have revealed that MFCs with diffuse arc resistance generally exhibit lower power densities. Feng et al. (2010) constructed an MFC with a total volume of 460 ml, and the MFC comprised a massive three-dimensional anode composed of graphite rods combined with activated carbon and an air cathode. The whole-cell Nyquist diagram demonstrated an oblique line at low frequencies (diffusion impedance). Paradoxically, the MFC impedance was low, which may have resulted from direct communication between the cathode and anode through activated carbon. In summary, irregular three-dimensional electrodes, such as large-scale MFCs and activated carbon anodes, are extremely likely to generate diffusion resistance (Wei et al., 2011). In addition, diffusion resistance is prone to occur when the cathode is limited by diffusion. This phenomenon may also occur in typical small-volume single-chamber air cathodes owing to the limitation of oxygen diffusion in the air cathode (Peng et al., 2013; Sevda et al., 2013; Lepage et al., 2014).

## Typical impedance equivalent circuit

The microbial electrode interface structure in MFCs is more complex than that in inorganic electrode interfaces; however, the structure should still be studied based on Randles. Figure 2A illustrates an equivalent circuit corresponding to a representative impedance plot. A few studies concluded that a pure electrode produced the response arc of all impedance spectra, and state variables such as adsorption on the electrode affected the current in the Faraday channel (Jung et al., 2011). Thus, the branch of the electrode electric double-layer capacitance was then connected in parallel with all other elements. As presented in Figure 2B(1), Li et al. (2014) connected the Cdl of the electric double-layer capacitance at the electrode–biofilm interface in series with the CPE and resistance RB, which reflected the dielectric properties of the biofilm, to study the extracellular electron transfer process under different polarization conditions. The lower part of the circuit represents the long-range electron transfer in a super-exchange manner, including the out-of-phase electron transfer resistance, Rct, between the electrode and redox groups, charge–discharge capacitance of the inner conductive layer of the biofilm caused by the potential disturbance, and charge exchange resistance, Rtr. The two branches of the circuit are arranged in parallel because they represent independent electrochemical processes. The resistance effect of the electrolyte is compensated for by the Rs element. Jung et al. (2012) used this circuit to assess the bioanode of an air cathode MFC and obtained two time constants: the high- and low-frequency arc corresponded to the anode electric double layer and other state variables of the bioanode, respectively [Figure 2B(2)].

In addition, some fitting circuits involve connecting the nonconductive branch in the Randles-electric double-layer capacitance and polarization internal resistance in parallel with other components in series; the number of components in series is equivalent to the number of multiple-time constants (Yin et al., 2013; Kircheva et al., 2015). Lepage et al. (2012) fitted the impedance of a reticulated foam carbon cathode to obtain the aforementioned series circuit structure. R1β represents the internal resistance related to electron transfer, and CPE1β denotes the electric double-layer capacitance under the diffusion effect (Figure 2C). Two time constants were obtained from the two peaks in the cathode Bode diagram. Because low frequencies have diffusion limitations, the circuit included diffusion impedance fitting. The authors argued that the complexity of the circuit simply reflects the complexity of the natural process in the MFC and that this circuit corresponds to the actual cathode process owing to the limitation of oxygen transport. The aforementioned study also used a generalized finite Warburg impedance to calculate the thickness (200–500 μm) of the cathode oxygen diffusion layer.

Ramasamy et al. (2009) used an MFC to investigate the endogenous mediator generation and discharge processes in *Shewanella oneidensis* MR-1. Riboflavin was added to reduce the low-frequency impedance; the experiment suggested that the low-frequency arc response reflected electron transfer during matrix oxidation. The experiment extended the impedance from the pure electrode to electron transfer in biological oxidation; therefore, the study adopted the series RQ circuit mode to equivalently change the space from electrode to biofilm. Lepage et al. (2014) believed that the common distribution of multiple time constants originated from the separation of an electric double layer and other conductive systems. Intuitively, they believed that microbial membranes were similar to organic coatings with separate conductive structures, such as oxide films, organic coatings, and human skin. For the bioanode, they chose a complex general distribution structure to represent the various effects of biofilm conduction. They expressed adsorption and diffusion using capacitance in the biofilm section. This model may be closer to the actual physical structure of a biofilm. Heijne et al. (2018) used EIS to quantify the biofilm capacitance. Instead of using a large capacitance electrode such as graphite, a planar electrode made of fluorinated tin oxide (FTO) was used for the anode in this experiment to separate the charge transfer, where different contributions of electrical, biofilm, diffusion resistance, and biofilm capacitance to anodic electrical response were clarified. The authors believe that the electrode capacitance in the EIS spectrum is of the same order of magnitude as the biofilm capacitance and can, therefore, be distinguished; however, separating the biofilm capacitance in electrode materials with a high capacitance is challenging. Using FTO as the electrode, arc separation, which represents the biofilm capacitance, can be observed in the Nyquist diagram as the electrode capacitance gradually decreases. Moreover, the authors discovered that the biofilm capacitance and current density increased with time, indicating that current and biofilm capacitance are closely related. When using the EIS fitting circuit depicted in Figure 2B(3), the circuit can effectively distinguish the electron transfer process occurring at the biofilm, electrolyte, and electrode interfaces. In addition, the model better represents the results of electrodes with a small capacitance.

## Effect of EIS capacitance characteristics on MFCs

**Figure 3:**
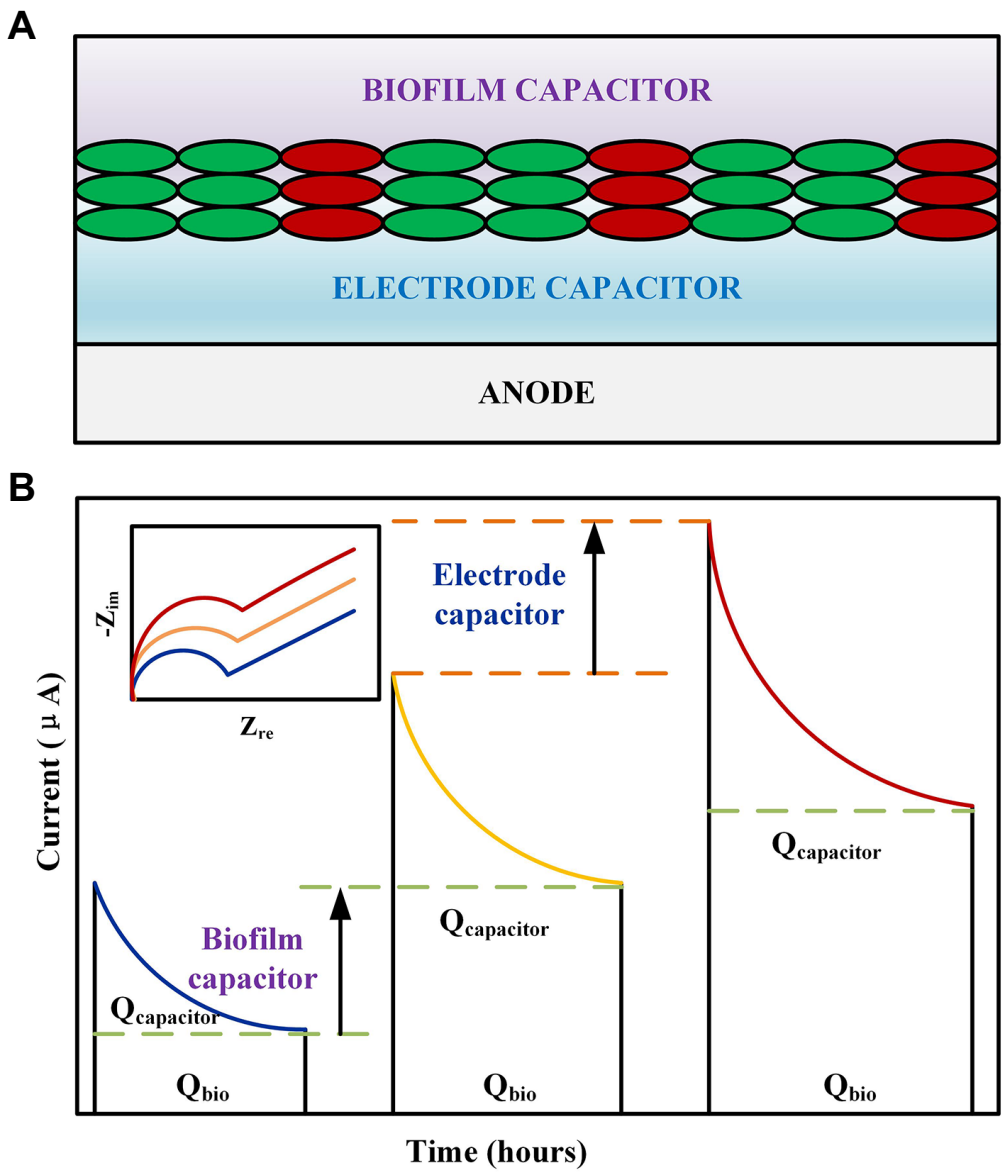
**(A)** Schematic of the representative capacitance of an MFC anode. **(B)** Effect of MFC electrode capacitance and biocapacitance on the discharge process. Inset: Nyquist diagram corresponding to the MFC electrode capacitance and biocapacitance.

In related research on MFCs, researchers have paid considerable attention to the real part of impedance, that is, the electron transfer, ohmic, and diffusion internal resistances (Zhang et al., 2011; Du et al., 2014). By analyzing the process and mechanism of the change in EIS under different conditions, the output power of the MFC was enhanced, and the removal effect of the MFC on pollutants was improved. However, limited research has been conducted on the origin and properties of the imaginary part of EIS, that is, the capacitance (MFC has almost no inductance). The source of electrode complex interfacial capacitance for biofilm attachment is believed to include electrode materials, cell bodies, and heme proteins that are responsible for electron transfer (Figure 3A; Ramasamy et al., 2009; Lu et al., 2015; Long et al., 2019). Recent studies have concluded that increasing the anode capacitance has a more significant effect on the stabilization and improvement of the current output (Figure 3). Carbon is a common electrode material with high capacitance. Deeke et al. (2012) discovered that the polymer material coatings of n-methyl-2-pyrrolidone, polyvinylidene fluoride, and carbon powder significantly improved the capacitance of the electrode material, thus increasing the MFC discharge current from 0.79 ± 0.03 to 1.02 ± 0.04 A/m^2^. Chen et al. (2018) prepared activated carbon/carbon nanotube supercapacitors for MFC anodes that achieved power densities of 899.52 and 555.10 mW/m^2^. Metallic materials, particularly manganese, are often used to improve the capacitive properties of materials. Zhang et al. (2015) electrodeposited manganese dioxide particles of different weights onto the surface of carbon felt for electrical performance experiments. The results revealed that depositing additional manganese dioxide resulted in a higher capacitance and helped stabilize the performance of the anode while enhancing the power output (3,580 ± 130 mW/m^2^, 24.7% higher than that of the carbon felt anode). Wang et al. (2016) used manganese dioxide-modified bioelectrodes in MFC owing to their good electron storage and release capabilities, which have proven to be beneficial for hexavalent chromium reduction by obtaining electrons at the cathode. An optimization of these characteristics is beneficial for the generation of biocapacitance by attaching microorganisms to the electrode surface (Figure 3B). This is because the improvement in material capacitance characteristics primarily corresponds to the improvement in specific surface area and pore structure adjustment. Li et al. (2022) used carbonized carbon fibers as bioanodes. The experiment revealed that the abundance of *Geobacter* attached to the carbonized material increased from 17 to 56%, corresponding to an increase in the biocapacitance from 1F to 3F, respectively; additionally, the power density increased by 70%. Fang et al. (Fang et al., 2022) investigated the effects of the thickness of the assembled *Shewanella* biofilm on the capacitance. The experiment concluded that although the total internal resistance increased from 680 to 1,000 Ω when the biofilm thickness had three layers, this thicker biofilm doubled the biofilm capacitance, corresponding to an increase in the power density from 13.3 to 29.2 μW/cm^2^. This demonstrated that the effect of capacitance on the current output was more pronounced than that of the resistance in certain specific cases.

Numerous studies have been conducted on the capacitance generated by microorganisms (biofilms). The primary consideration therein is the CPE parameter α. Lu et al. (2015) believed that cells had a capacitive structure-phospholipid bilayer causing the biocapacitance to increase with the number of cells. The anode capacitance of the MFC markedly increased with an accompanying decrease in the resistance over 12 days. Active substances such as electron shuttles, nanowires, and cytochrome c were also found to be related to the biocapacitance. Accordingly, the following two formulas are used to express the relationship between capacitance and resistance in the growth stage of microorganisms:


(7) $$C=K\varepsilon \times \frac{NAS}{4\pi kd}$$

where C denotes the capacitance of the anode; K represents a constant; N denotes the number of anode electricity-producing bacteria; A represents the number of electron transporters per electricity-producing bacteria, that is, the number of cytochrome c and nanowires; ε corresponds to the dielectric constant; and d refers to the capacitor plate spacing.


(8) $$R_{a}=K^{\prime }\times \frac{1}{NAS}$$

where $R_{a}$ represents the anode resistance, and $K^{\prime }$ denotes a constant.

In the growth process of microorganisms, the capacitance of the bioanode is proportional to the quantity of active substances covered by the microorganisms, whereas the resistance is inversely proportional to the capacitance, and the product of the capacitance and resistance is constant. Thus, the following formula is obtained:


(9) $$CR_{a}=KK^{\prime }\varepsilon \times \frac{1}{4\pi Kd}$$

Experiments revealed that the capacitance multiplied by the internal resistance of the anode during the microbial growth stage is a constant value (Lu et al., 2015). It is established that electrodes used in MFC research are unpolished with rough surfaces and non-uniform three-dimensional directions. Therefore, a CPE is often used to describe the microbial capacitance. Lepage et al. (2014) studied the electrochemical response of an MFC at different operating temperatures (15–25°C), phosphate buffer solution concentrations (4–8 mM), acetic acid concentrations (7.1–14.3 mM), and equivalent solution conductivities (2.5–5 mS/cm). The authors used a deterministic model to simulate an effective circuit and plotted the value of the imaginary part of the impedance, Zim, against the frequency to obtain the slope and, thus, determine the CPE parameter α. The following formula can be used to describe the charge accumulation corresponding to the effective capacitance:


(10) $$C=\mathrm{gQ}\left(\rho_{0}\varepsilon \varepsilon_{0}{}^{1-\alpha }\right)$$

(11) g=1+2.88(1−α)2.375

where α denotes Constant Phase Element (CPE) parameter; *Q* represents the pseudo-capacitive CPE parameter; *ε_0_* is the permittivity of vacuum; *ρ_0_* is the boundary value of resistivity at the interface; and *g* is a numerically evaluated function:

Experiments revealed that α varied between 0.36 and 0.93; this variation was primarily caused by α and microbial adsorption through equivalent circuit fitting (Lepage et al., 2014). Moreover, the same formula was used to assess the influence of the external resistance and hydraulic retention time on the impedance of MFC electrodes and whole-cells and to analyze the arc corresponding to multiple frequencies and the parameter α (Sevda et al., 2015). For the low-frequency response of the bioanode (related to the internal transfer of electrons or generation of electrons from substance oxidation), α increased with an increase in the external resistance (0.7–0.85). Owing to the increase in external resistance, the process of electron transfer to the cathode was more resistant, resulting in an accumulation of electrons in the form of an electric double layer at the anode and an enhanced uniformity of the electrode characteristics. The α value of the cathode remained stable in the low-frequency response at approximately 0.58, even when the external resistance changed. This indicated that the change in external resistance limited the bioanode reaction process, which, in turn, affected the power generation performance of the MFC. In the high-frequency region, α was considered irrelevant to the rate limit and decreased from 0.7 to 0.4. The authors propose the concept of confinement film through effective capacitance; however, the confinement film does not correspond to a specific structure, which complicates the analysis of the MFC capacitance.

In general, the α value of a bioanode increases with time. Aaron et al. (2010) determined the trend of pseudocapacitance α with time; α increased from 0.124 to 0.975, whereas the cathode capacitance remained almost unchanged. They believed that a biological action reduced the electrode inhomogeneity, which was confirmed by Borole et al. (2010). However, the opposite is true for the corresponding capacitance. The pseudocapacitance calculated using the aforementioned formula indicated that the capacitance was significantly reduced. The results provided by Borole et al. (2010) revealed that the capacitance increased from 0.007 to 0.45 with an increase in the biofilm culture time. At present, the equation for calculating the effective biocapacitance is satisfactory, and the results of the equation 10 can be compared using galvanostatic charge–discharge and cyclic voltammetry to obtain better results.

## An EIS analysis method for bioelectrode: Distribution of relaxation times

In general, the total number of physicochemical processes that determine a particular system under study is not known *a priori*; therefore, provisional assumptions based on physical intuition must be made. For most electrochemical systems, discrete process time constants tend to overlap, and assignments to qualified models can become ambiguous (Schlüter et al., 2019). Most EIS plot can be fitted using multiple equivalent circuits; therefore, the choice of circuit often depends on the experience of the researcher. DRT can deconvolve the impedance data and convert the data into a distribution of time constants, which can effectively identify electrochemical processes of a similar scale (Weiß et al., 2017; Melo et al., 2021). This could render DRT a powerful tool for analyzing bioelectrode. Owing to the so-called “ill-posed problem” of inversion, DRT cannot be assumed to be correct, and the use of different inversion methods may lead to a loss of data. Therefore, results obtained from the inversion and original data should be verified; this verification process is complex and time-consuming (Dierickx et al., 2020). Thus, DRT has not been widely used in MFC measurements. Currently, auxiliary tools are available for DRT verification that can enhance the usefulness of DRT to a certain extent. Melo et al. (2021) constructed a tool that converted the DRT data into a synthetic impedance spectra, which could then be compared with the original measured impedance data to resolve the difference in DRT results originating from different inversion methods. This tool ensures that the results obtained from the inversion are consistent with the original data and calculates the resistance, time constant, and capacitance of each response satisfactorily. With the aid of such tools, DRT can be better applied to analyze indistinguishable data, which is extremely helpful for analyzing the capacitance of MFC biofilms.

**Figure 4:**
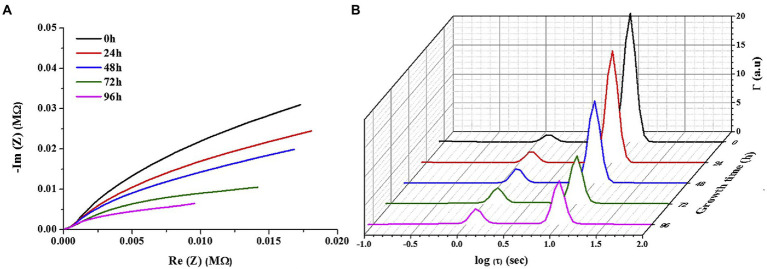
**(A)** Representative Nyquist plot for bacterial growth time, and **(B)** representative DRT for increasing growth time (Bharatula et al., 2020).

EIS has been widely used for the detection of powerfully electroactive biofilms. The ability of EIS to detect the impedance of weakly electroactive microorganisms has increased with the development of DRT tools. As presented in Figure 4, Bharatula et al. (2020) used *Pseudomonas aeruginosa* as the research object and indium tin oxide-coated polyethylene terephthalate as the carrier. After EIS measurements and DRT analysis, they discovered that the time constants dominated by biofilm impedance were approximately 1 and 0.01 s (Figure 4B). Therefore, the authors adopted an equivalent circuit with two-time constants. A Randles-equivalent circuit using two CPE elements in the circuit represented the electrochemical system, and another resistor in series represented the resistance of the solution. The equivalent circuit could represent the electrode–biofilm and electrode/biofilm–solution interfaces and solution behavior. This is the most common fitting circuit, and it was verified to be applicable for the fitting of weakly electroactive biofilms. DRT has gradually emerged as an important tool for EIS with the development of auxiliary tools. In the near future, this advanced inversion technique is expected to play a significant role in the practical application of MFCs, thereby facilitating their advancement.

## Conclusion

While EIS is powerful for investigating the impedance of MFCs, an appropriate interpretation of the physical structures of complex biofilms using EIS results is still challenging. In this review, we discuss how to choose the model to fit the EIS data under suitable measuring conditions, and introduce the emerging DRT tool to effectively identify electrochemical processes. Meanwhile, the importance of capacitance to MFC performance is collectively reviewed, which could be used to evaluate the material synthesis for the bioelectrode. In addition to the usage of EIS to investigate model microbial fuel cell, EIS is applied to large-scale MFCs, such as constructed wetland and sediment MFCs, and other bioelectrochemical processes, such as microbial induced corrosion, and microbial desalination cells.

## Author contributions

HW and XL contributed to conception and design of the study. YS, DW, and ZW wrote sections of the manuscript. HM, CJ, WD, and NL drew these pictures and checked the language. All authors contributed to manuscript revision, read, and approved the submitted version.

## Funding

This work was supported by the National Natural Science Foundation of China (42107030, 52070156), Technology Innovation Center for Land Engineering and Human Settlements, Shaanxi Land Engineering Construction Group Co., Ltd., and Xi’an Jiaotong University (2021WHZ0094), Natural Science Basic Research Program of Shaanxi Province (2021JM-329), Natural Science Foundation of Shaanxi Provincial Department of Education (20JK0783), and the Postdoctoral Program from Japan Society for the Promotion of Science (P20105).

## Conflict of interest

YS and NL were employed by the company Shaanxi Land Engineering Construction Group Co., Ltd.

The remaining authors declare that the research was conducted in the absence of any commercial or financial relationships that could be construed as a potential conflict of interest.

## Publisher’s note

All claims expressed in this article are solely those of the authors and do not necessarily represent those of their affiliated organizations, or those of the publisher, the editors and the reviewers. Any product that may be evaluated in this article, or claim that may be made by its manufacturer, is not guaranteed or endorsed by the publisher.
